# Black raspberries attenuate colonic adenoma development in *Apc*^*Min*^ mice: Relationship to hypomethylation of promoters and gene bodies

**DOI:** 10.1002/fft2.45

**Published:** 2020-09-09

**Authors:** Yi-Wen Huang, Yue Yang Mo, Carla Elena Echeveste, Kiyoko Oshima, Jianying Zhang, Martha Yearsley, Chien-Wei Lin, Jianhua Yu, Pengyuan Liu, Ming Du, Chongde Sun, Jianbo Xiao, Li-Shu Wang

**Affiliations:** 1 Department of Obstetrics and Gynecology, Medical College of Wisconsin, Milwaukee, Wisconsin; 2 Division of Hematology and Oncology, Department of Medicine, Medical College of Wisconsin, Milwaukee, Wisconsin; 3 Department of Pathology, Johns Hopkins University, Baltimore, Maryland; 4 Division of Biostatistics, Department of Science of Informatics, City of Hope National Medical Center and Beckman Research Institute, Duarte, California; 5 Department of Pathology, The Ohio State University, Columbus, Ohio; 6 Division of Biostatistics, Medical College of Wisconsin, Milwaukee, Wisconsin; 7 Department of Hematology & Hematopoietic Cell Transplantation, City of Hope National Medical Center and Beckman Research Institute, Duarte, California; 8 Center of Systems Molecular Medicine, Department of Physiology, Medical College of Wisconsin, Milwaukee, Wisconsin; 9 Sir Run Run Shaw Hospital and Institute of Translational MedicineZhejiang University, Zhejiang, China; 10 School of Food Science and Technology, National Engineering Research Center of Seafood, Dalian Polytechnic University, Dalian, China; 11 Laboratory of Fruit Quality Biology / Zhejiang Provincial Key Laboratory of Horticultural Plant Integrative Biology / The State Agriculture Ministry Laboratory of Horticultural Plant Growth, Development and Quality Improvement, Zhejiang University, Zijingang Campus, Hangzhou, China; 12 State Key Laboratory of Quality Research in Chinese Medicine, Institute of Chinese Medical SciencesUniversity of Macau, Taipa, Macau, China

**Keywords:** functional foods, healthy food, nutrition

## Abstract

Recent studies have suggested that in addition to promoter region, DNA methylation in intragenic and intergenic regions also changes during physiological processes and disease. The current study showed that feeding of black raspberries (BRBs) to *Apc*^*Min*^ mice suppressed colon and intestinal tumors. MBDCap-seq suggested that dietary BRBs hypomethylated promoter, intragenic, and intergenic regions. Annotation of those regions highlighted genes in pathways involved in immune regulation, inflammatory signaling, production of nitric oxide and reactive oxygen species, and progression of colorectal cancer. BRB phytochemicals (e.g., ellagic acid, anthocyanins, oligosaccharides) and their gut bacterial metabolites (e.g., urolithin, protocatechuic acid, short-chain fatty acids) inhibited DNMT1 and DNMT3B activities in a cell-free assay. Our results suggest that BRBs’ hypomethylating activities result from the combined effects of multiple BRB phytochemicals and their gut bacterial metabolites. Because similar substances are found in many plant products, our results with BRBs might also apply to commonly consumed fruits and vegetables.

## 1 | INTRODUCTION

Epigenetic gene silencing generally encompasses three related processes: DNA methylation, histone modification, and chromatin remodeling (Huang, Kuo, Stoner, Huang, & Wang, 2011), and it has been linked to multiple human clinical disorders including cancers (Hongwei, Haixia, Daidi, & Jianjun, 2020; Pan et al., 2017a, 2018, 2020; Wang et al., 2013b; Wang, Kuo, Huang, Stoner, & Lechner, 2012), Huntington’s disease (Khan et al., 2020), diabetes mellitus (Chao, Xuzhi, Sheng, & Hui, 2020), etc. The most studied of these three is DNA methylation, which is regulated by DNA methyltransferases (DNMTs) such as DNMT1 and DNMT3B (Baylin, 2002; Herman & Baylin, 2003). However, methylation alone may not initiate gene silencing and itself does not directly repress transcription because the composition of the chromatin surrounding a hypermethylated gene promoter contributes to that gene’s functional state (Baylin, 2002; Herman & Baylin, 2003). Thus in cancer, tumor suppressor genes are silenced by interactions between DNA hypermethylation and inactivated chromatin (Cui et al., 2014). Although histone deacetylase (HDAC) inhibitors robustly reexpress silenced genes without changing a promoter’s methylation status, the removal of DNA methylation signals is required to achieve long-term gene reactivation (Raynal et al., 2012; Cui et al., 2014). These findings define chromatin as an important druggable target for cancer prevention/therapy (Cui et al., 2014). In fact, treatment with decitabine (targeting DNMTs) in combination with panitumumab (targeting epidermal growth factor receptor) stabilized disease in 10 of 20 metastatic colorectal cancer patients in a phase I/II study (Garrido-Laguna et al., 2013).

We used the *Apc*^*Min*^ mouse in the current study, as mutations in the Apc (adenomatous polyposis coli) gene in the Wnt signaling pathway are considered gatekeeper mutations in colorectal cancer. For example, an inherited mutation in Apc results in familial adenomatous polyposis (FAP). Apc is also mutated in up to 80% of all sporadic colorectal tumors (Fenton & Hord, 2006). Historically, the *Apc*^*Min*^ mouse model has been used extensively to investigate mechanisms of colon cancer, although it develops tumors predominately in the small intestine. Recent evidence suggests, however, that tumors in the colon of *Apc*^*Min*^ mice are pathologically and molecularly similar to human colon adenocarcinomas. For example, overexpression of the DNMT3B1 led to a twofold increase in colon tumors in *Apc*^*Min*^ mice and increased the average size of colonic microadenomas (Linhart et al., 2007). This is relevant to human colon cancer because methylation of genes by DNMT3B1 in the *Apc*^*Min*^ mouse colon closely resembles the de novo methylation reported for human colorectal cancer (Steine et al., 2011). Also, *Apc*^*Min*^ mice carrying a CDX2P-NLS Cre recombinase transgene and a loxP-targeted Apc allele developed mainly colorectal tumors (Hinoi et al., 2007). Biomarkers of metabolic reprogramming in colon tumors from these mice were similar to biomarkers in human colorectal cancer (Manna et al., 2014). Thus, while there are no perfect models for human colorectal cancer, the *Apc*^*Min*^ model incorporates mutations that elicit phenotypic responses similar to those in humans (Tammariello & Milner, 2010).

In pioneering investigations, our group studied the effects of freeze-dried black raspberries (BRBs) on colorectal cancer. In 20 patients who consumed BRBs for an average of 4 weeks, we found that tumor suppressor genes were demethylated by BRBs and they modulated biomarkers in the colon and rectum cancer development. Demethylation associated with changes in DNMT1 levels (Wang et al., 2011). In patients with FAP, daily treatment with BRB rectal suppositories for 9 months led to regression of 36% of rectal polyps. Also, the BRBs demethylated more promoter regions in polyps from the responders than in those from the nonresponders (whose tumors did not regress). Thus, it might be possible that nonresponsiveness is due to their decreased responses to DNA hypomethylation induced by BRBs (Wang et al., 2014). We further reported that anthocyanins in BRBs decreased the activity of DNMT1 and were uptaken into HCT116 cells, where anthocyanins and DNMT1 localized at different spots (Wang et al., 2013a). Similar results were observed for DNMT3B when the cells were treated with BRB anthocyanins. These findings suggest that anthocyanins indirectly regulate DNMT1 and DNMT3B and are responsible for whole BRBs’ demethylation effects in colorectal cancer. Because ulcerative colitis is a precursor to colorectal cancer, we also determined if the anti-inflammatory effects of BRBs associated with their hypomethylation activities. We reported that BRBs suppressed colonic inflammation by hypomethylating promoter methylation of Wnt pathway suppressors through regulating DNMT1 and DNMT3B in IL-10 knockout mice (Wang et al., 2013c) and dextran sodium sulfate-induced colonic ulceration in mice (Wang et al., 2013d).

The current study used MBDCap-seq to investigate the genome-wide hypomethylation effects of BRBs in the colon of *Apc*^*Min*^ mice. We found that feeding BRBs led to hypomethylation in promoter, intragenic, and intergenic regions annotated to genes in pathways that regulate the immune system, inflammation, and colorectal cancer progression in these mice. Phytochemicals found in BRBs, such as ellagic acid, anthocyanins, and oligosaccharides, are able to suppress the activities of DNMT1 and DNMT3B, as can gut bacterial metabolites of these phytochemicals, such as urolithin A, urolithin B, protocatechuic acid, and short-chain fatty acids. Our results therefore suggest that both BRB phytochemicals and their metabolites generated by gut bacteria contribute to the hypomethylation effects of whole BRBs.

## 2 | MATERIALS AND METHODS

### 2.1 | Animals and BRB treatment

All protocols in this study were carried out according to the Institutional Animal Care and Use Committee (IACUC) guidelines for animal care at the Medical College of Wisconsin (protocol approval number: AUA2430; initial approval date: August 26, 2011; expiration date: April 14, 2023). BRB treatments were described before in our previous publication (Wang et al., 2013b). Three to four week-old wild-type and *Apc*^*Min*^ male and female breeder mice were purchased from The Jackson Laboratory (Bar Harbor, ME). One week later, all experimental animals were placed on a control diet (male, *n* = 15; female, *n* = 15) or a 5% BRB diet (male, *n* = 15; female, *n* = 15). After 10 weeks on those diets, the mice were sacrificed, and mucosal layers from colon and small intestine were collected and snap-frozen in liquid nitrogen (*n* = 10 per group). In addition, the entire colon and small intestine were formalin-fixed and paraffin-embedded (*n* = 5 per group).

### 2.2 | Real-time PCR

mRNA from the snap-frozen mucosa was extracted, using RNeasy Mini Kit (Qiagen, Valencia, CA). Two micrograms of total RNA per sample was reverse transcribed, using Superscript III RT (Invitrogen, Waltham, MA). For mouse TLR-4, MYD-88, and IRAK3 primers, they were purchased from Integrated DNA Technologies (Coralville, IA). These primers are commercially available, and they were validated by Integrated DNA Technologies (Coralville, IA). A gene’s expression was compared to its threshold cycle (Ct) with the Ct of the housekeeping gene GAPDH.

### 2.3 | Bisulfite pyrosequencing

DNA from mucosa of colon and small intestine from the wild-type and *Apc*^*Min*^ mice fed a control or 5% BRB diet was isolated using the PicoPure DNA kit (MDS Analytical Technologies, Sunnyvale, CA). Then isolated DNA was purified using the QIAquick PCR purification kit (Qiagen, Valencia, CA). Five-hundred ng of those DNA was bisulfite-converted by the EZ DNA Methylation kit (Zymo Research, Irvine, CA). Methylated CpGs in the promoter regions of SFRP1, DKK3, and IRAK3 were quantified by a pyrosequencing system (Qiagen, Valencia, CA) as described previously (Wang et al., 2013c).

### 2.4 | Immunohistochemical staining and computer-assisted image analysis

Colon and small intestine from wild-type and *Apc*^*Min*^ mice fed the control or 5% BRB diet were formalin fixed and paraffin embedded, and then they were cut into 4-*μ*m sections and placed on slides. Staining and quantification of nuclear stained *β*-catenin and NF*κ*B were described in our earlier publication (Wang et al., 2013d). The commercial sources of *β*-catenin and NF*κ*B p65 antibodies from Cell Signaling Technology (Danvers, MA). Stained slides were scanned, and 40× imagines were taken for analysis of nuclear staining, using Simple PCI (HCImage, Sewickley, PA).

### 2.5 | DNMT1 and DNMT3B inhibition assays

EpiQuik DNA Methyltransferase 1 kit and a 3B Activity/Inhibitor Screening Assay kit (Epigentek, Brooklyn, NY) were used to access inhibition of DNMT1 and DNMT3B, respectively. Ellagic acid, urolithin A, urolithin B, cyanidin-glucoside, cyanidin-rutinoside, protocatechuic acid, fructo-oligosaccharides, galacto-oligosaccharides, xylo-oligosaccharides, butyric acid, acetic acid, propionic acid, and valenic acid were purchased from SigmaAldrich (St. Louis, MO). Each compound was dissolved in dimethyl sulfoxide to prepare stock solution, then diluted to 1 nM, 100 nM, and 10 *μ*M and tested in DNMT1 and DNMT3B inhibition assays. Each compound was tested in both assays in three independent runs.

### 2.6 | MBDCap-seq for genome-wide DNA methylation analysis

Snap-frozen colon mucosa from *Apc*^*Min*^ mice on the control diet (*n* = 5) or BRB diet (*n* = 5) were used for MBDCap-seq, following the manufacturer’s protocol (MethylMiner, Invitrogen), as described in our previous publication (Wang et al., 2014). In briefly, genomic DNA (1 *μ*g) was sheared by sonication into 200–600-bp fragments, and methylated DNA was immuno-precipitated by incubating of sonicated genomic DNA for 1 hr at room temperature with 3.5 *μ*g of recombinant MBD-biotin protein and Streptavidin beads. Methylated DNA was eluted with high-salt buffers (1 Mmol/L NaCl), and then recovered by ethanol precipitation procedure. The DNA fractions were subjected to library generation and followed by an Illumina genome analyzer.

### 2.7 | Analysis of MBDCap-seq DNA methylation data

MBDCap-seq, mapping, and normalization were was analyzed by BELT (Bin-based Enrichment Level Threshold) as described in our earlier publication (Wang et al., 2014). Briefly, the BELT algorithm includes four steps: (1) define a series of bin size by evenly dividing the genome varying from 100 to 300 bp and counting the density of reads for each bin. It should be noted that the methylated regions can be any length but 8 kb was used, and the reason was that the majority of CpG islands are within 2 kb upstream or downstream of the transcription start site. CpG island shores are up to 2 kb away relative to each gene’s CpG islands; (2) determine significant enrichment threshold levels by a percentile rank statistic method; and (3) estimate false discovery rates by utilizing Monte Carlo simulation for modeling background based on signal-noise-ratio of MBDCap-seq data. BELT uses a control dataset, such as IgG or input sequence reads, and utilizes a Fisher exact test to compute the *p*-value for identified peaks. Ingenuity pathway analysis was used for signaling analysis.

### 2.8 | Statistical analysis

GraphPad Prism was utilized to analyze tumor numbers and sizes, mRNA levels, and methylation percentages determined by bisulfite pyrosequencing (unpaired, two-tailed *t*-test). A *p*-value <.05 was considered statistically significant.

## 3 | RESULTS AND DISCUSSION

### 3.1 | BRBs suppressed tumor progression in colon and small intestine in male and female *Apc*^*Min*^ mice

**FIGURE 1:**
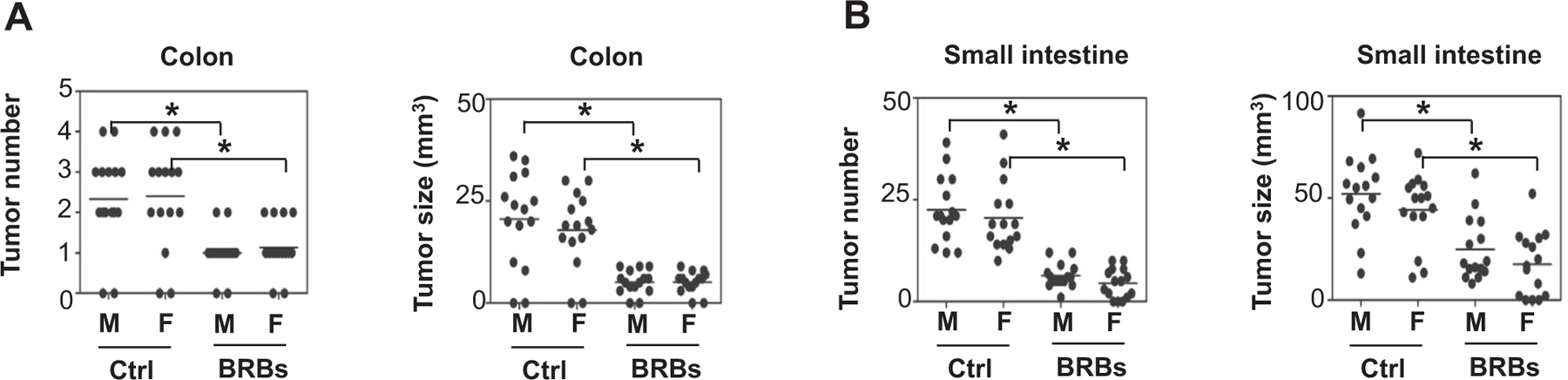
BRBs suppressed tumor progression in colon and small intestine in male and female *Apc*^*Min*^ mice. At 4–5 weeks of age, male and female mice were fed control or BRB diets for 10 weeks. Then the number of tumors in colon (A) and small intestine (B) were counted, and their sizes were measured. M: male; F: female; Ctrl: AIN-76A diet; BRBs: 5% black raspberries. Control diet (male, *n* = 15; female, *n* = 15) or BRB diet (male, *n* = 15; female, *n* = 15). *p < .05

Evidence has suggested that male and female *Apc*^*Min*^ mice respond differently to genetic modifications because deletion of Cox2 gene in intestinal epithelial cells decreases tumorigenesis in female but not male *Apc*^*Min*^ mice (Cherukuri et al., 2014). Also, two independent groups of scientists reported that exercise-induced attenuation of intestinal polyp development differed between genders (Colbert et al., 2003; Mehl et al., 2005). In order to determine if male and female Min mice respond differently to BRBs, 3–4-week-old *Apc*^*Min*^ mice initially tumor-free were fed control diet (AIN-76A) or a BRB diet. After 10 weeks, we sacrificed the animals. There was no difference in tumor number or size in the colon (Figure 1A) or small intestine (Figure 1B) between the male and female mice. Also, BRBs were equally effective at decreasing tumor number and size in colon (Figure 1A) and small intestine (Figure 1B) from both genders, suggesting that male and female Min mice respond similarly to BRBs, as we reported earlier (Pan et al., 2017d).

### 3.2 | BRBs suppressed tumor progression in colon and small intestine of *Apc*^*Min*^ mice by regulating the methylation of genes in the Wnt and TLR4 pathways

The toll-like receptor (TLR) family represents a critical part of innate immune recognition (Wells, Rossi, Meijerink, & van Baarlen, 2011). In particular, TLR4 recognizes lipopolysaccharide from the outer membrane of Gram-negative bacteria, the most common type of colonic bacteria (Poxton, Brown, Sawyerr, & Ferguson, 1997). TLR4 is overexpressed in human colorectal cancer tissues; increasing TLR4 expression is seen with advancing tumor stages and decreased overall survival in human colorectal cancer (Sussman et al., 2014). TLR4 can trigger a neoplastic program through activation of the Wnt pathway in villin-TLR4 mice treated with azoxymethane (AOM) (Santaolalla et al., 2013). These studies suggest that TLR4 functions as a tumor promoter in colorectal cancer.

**FIGURE 2:**
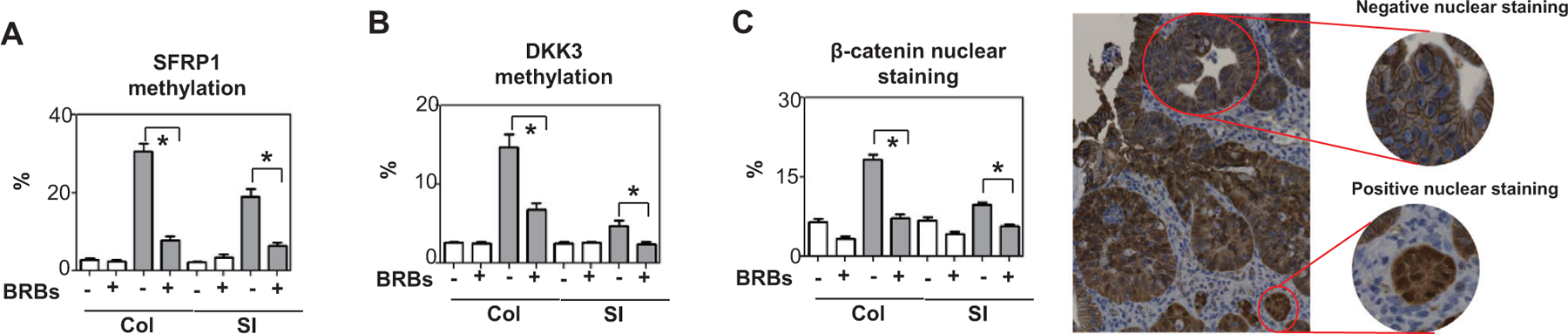
BRBs inhibited Wnt signaling in colon and small intestine of *Apc*^*Min*^ mice. BRBs decreased promoter methylation of (A) SFRP1 and (B) DKK3 as well as *β*-catenin nuclear staining (C). White bars: wild-type mice; gray bars: *Apc*_*Min*_ mice. Col: colon; SI: small intestine. *N* = 5 per group. *p < .05

**FIGURE 3:**
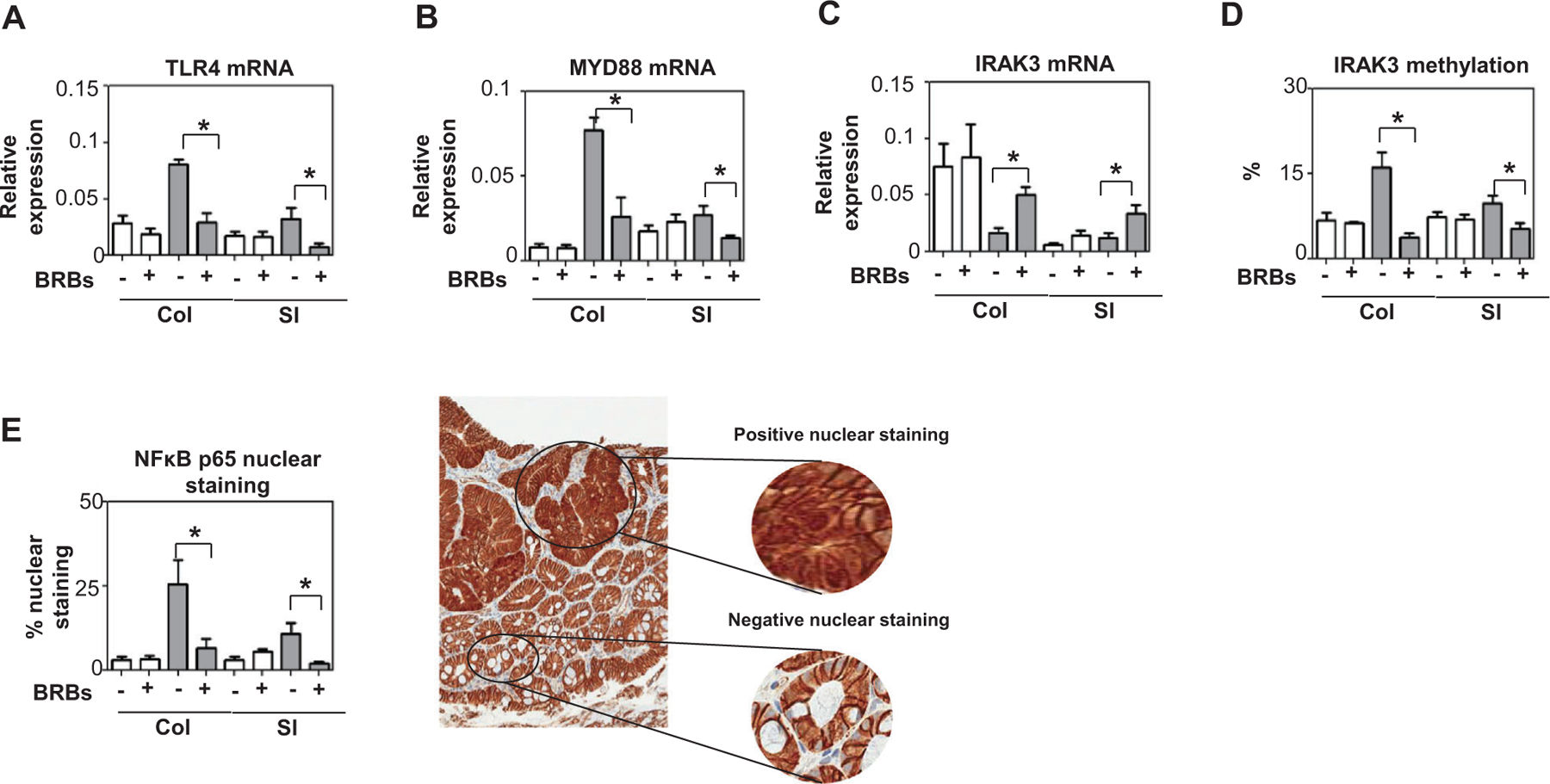
BRBs inhibited TLR4 signaling in colon and small intestine of *Apc*^*Min*^ mice. BRB feeding decreased mRNA levels of (A) TLR4 and (B) MYD88. Increased levels of IRAK3 mRNA (C) were accompanied by decreased promoter methylation (D). BRBs also decreased NF*κ*B nuclear staining. White bars: wild-type mice; gray bars: *Apc*^*Min*^ mice. Col: colon; SI: small intestine. N = 5 per group. *p <.05

In our studies of the Wnt and TLR4 pathways, we used colon and small intestine from male mice (as we had observed no gender differences in response to BRBs; Figure 1). Promoter methylation of SFRP1 and DKK3 (Wnt pathway antagonists) was higher in the Min mice than the wild-type mice (Figure 2). In these mice, BRBs decreased promoter methylation of SFRP1 and DKK3 and lessened *β*-catenin activation (Figure 2). In addition, BRBs suppressed hyper-TLR4 signaling, as evidenced by decreased levels of TLR4 and NF*κ*B (Figure 3). They also decreased promoter methylation of IRAK3, a TLR4 pathway antagonist, enhancing its expression (Figure 3). Our results suggest that BRB feeding hypomethylates tumor suppressors in the Wnt and TLR4 pathways, in turn attenuating *β*-catenin and NF*κ*B activation. In an ulcerative colitis model induced by 1,2‑dimethyl hydrazine and dextran sodium sulfate in ICR mice, colitis‑associated tumorigenesis decreased levels of IRAK‑3, suggesting it is anti-inflammatory (Xu, Zhang, Wang, & Chen, 2017). Hyper-TLR4 signaling in the colon and small intestine of the Min mice suggests that inflammation contributes to tumor development in those animals (Figure 3). Therefore, it is likely that TLR4 activates the Wnt pathway (Santaolalla et al., 2013). Our results suggest that BRBs play an anti-inflammatory role when they suppress colonic and intestinal tumors in the Min mice.

### 3.3 | MBDCap-Seq identified other pathways hypomethylated by BRBs

Transcription of DNA into RNA begins at the transcriptional start sites of genes’ promoter regions (Herman & Baylin, 2003). Mounting evidence suggests that methylation of promoter regions generally correlates with silenced tumor-suppressor genes (Herman & Baylin, 2003). However, recent studies have suggested that methylation in intragenic and intergenic regions also change during physiological processes and disease (Kulis, Queirós, Beekman, & Martín-Subero, 2013). Such studies are undergoing active investigation ( Jiang, Liu, Chen, Cao, & Tao, 2013; Maunakea et al., 2010). Interestingly, CpG islands in promoter, intragenic, and intergenic regions all overlapped with RNA markers of transcription initiation. Unmethylated CpG islands also overlapped significantly with trimethylation of H3K4, a histone modification that is enriched at promoters (Maunakea et al., 2010). This study further suggests that intragenic methylation plays a major role in regulating cell-specific alternative promoters in gene bodies (Maunakea et al., 2010).

**FIGURE 4:**
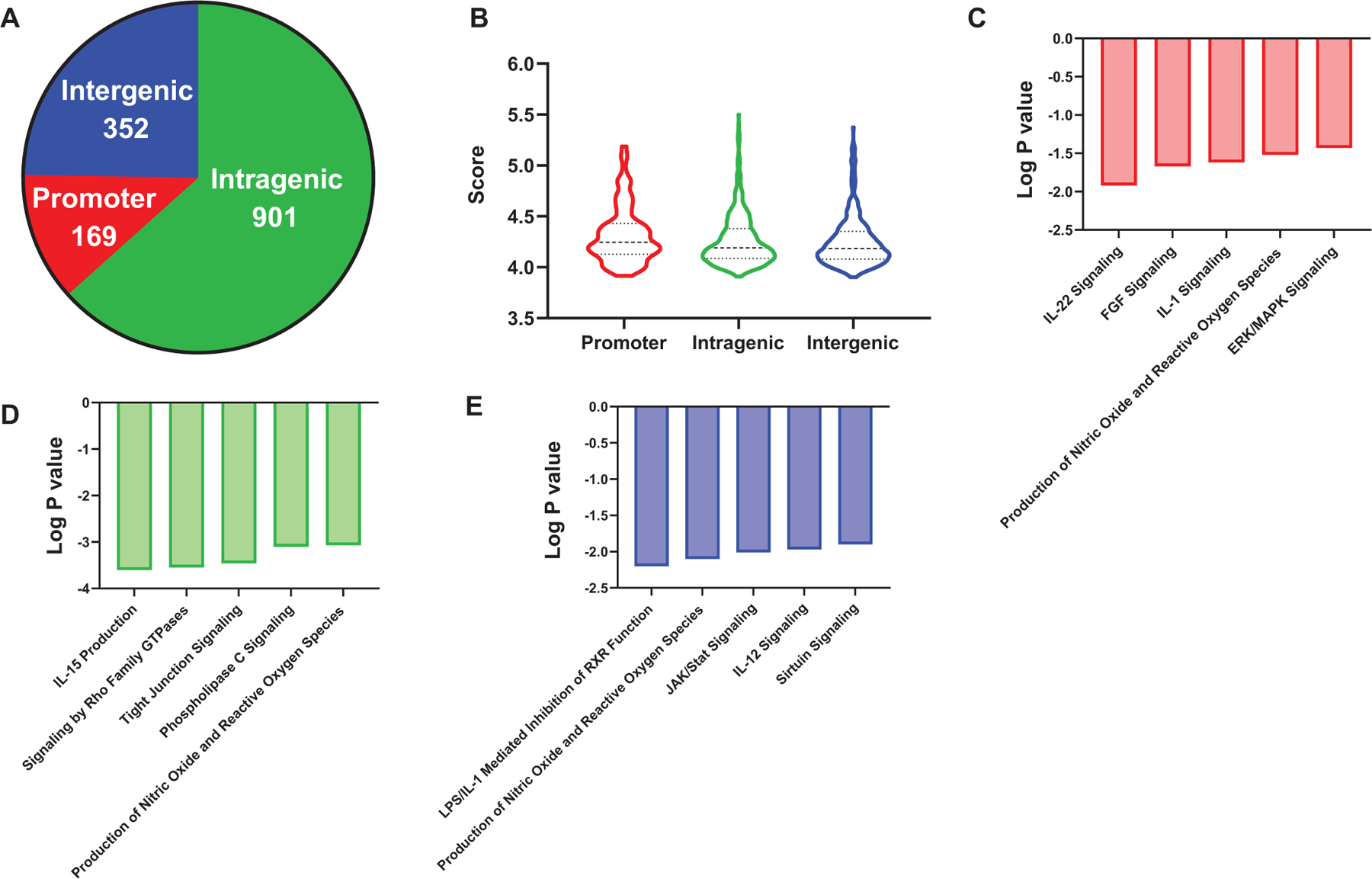
MBDCap-seq identified pathways hypomethylated by BRBs in the colon of *Apc*^*Min*^ mice. (A) Number of regions hypomethylated by BRBs in promoter, intragenic, and intergenic regions. (B) Methylation scores of those three regions. Pathway analysis using genes hypomethylated in promoter (C), intragenic (D), or intergenic (E) regions. Five of the top 10 pathways relevant to colorectal cancer are shown for each region. (*N* = 5 per group)

We did not previously explore the hypomethylation effects of BRBs in intragenic and intergenic regions (Wang et al., 2014). In the current study, MBDCap-seq was utilized to investigate their genome-wide hypomethylation effects in colon of *Apc*^*Min*^ mice. In total, 1422 regions were significantly hypomethylated. Most were located in intragenic regions followed by intergenic and promoter regions (Figure 4A). The extent of demethylation did not differ significantly among those three regions (Figure 4B). However, pathway analysis of the hypomethylated regions in all three regions identified multiple cytokine signaling pathways that regulate the immune system and inflammation (Figure 4C–E). In addition, genes in pathways involved in controlling colorectal cancer progression were demethylated by BRBs in all three regions (Figure 4C–E), as was pathway that produce nitric oxide and reactive oxygen species (Figure 4C–4E). Although BRBs led to hypomethylation of different pathways in promoter, intergenic, and intragenic regions, the current study suggests that all three regions contribute to BRBs’ antitumorigenic effects on the immune system, inflammation, and the production of nitric oxide and reactive oxygen species.

### 3.4 | BRB phytochemicals and their gut bacterial metabolites suppressed activities of DNMT1 and DNMT3B

DNMT1, DNMT3A, and DNMT3B, belonging to a family of highly related DNA methyltransferase enzymes, transfer methyl groups from S-adenosylmethionine onto the 5′ position of cytosine bases in the dinucleotide sequence CpG. This modification controls DNA methylation in mammalian cells (Herman & Baylin, 2003). DNMT1 functions to accurately replicate genomic patterns of DNA methylation during the S phase of the cell cycle, and therefore is named the maintenance DNA methyltransferase (Bestor, 2000). On the other hand, DNMT3A and DNMT3B enzymes are believed to have both activities of maintenance and de novo DNA methylation (Okano, Xie, & Li, 1998). However, DNMT1, DNMT3A, and DNMT3B all have some level of both activities of maintenance and de novo methylation in vitro (Robertson et al., 1999). Interestingly, the three DNMTs are overexpressed in several tumor types, for example, colorectal, bladder, and kidney (Robertson et al., 1999). A great example of how these enzymes control the expression of tumor suppressor genes is that methylation of CDKN2A is almost entirely eliminated and re-expressed when both DNMT1 and DNMT3B are deleted in cells lines of colon cancer (Rhee et al., 2002). Accordingly, aberrant methylation of tumor suppressor genes mediated by DNMTs represents an encouraging target for both chemoprevention and chemotherapy (Issa, 2007).

**FIGURE 5:**
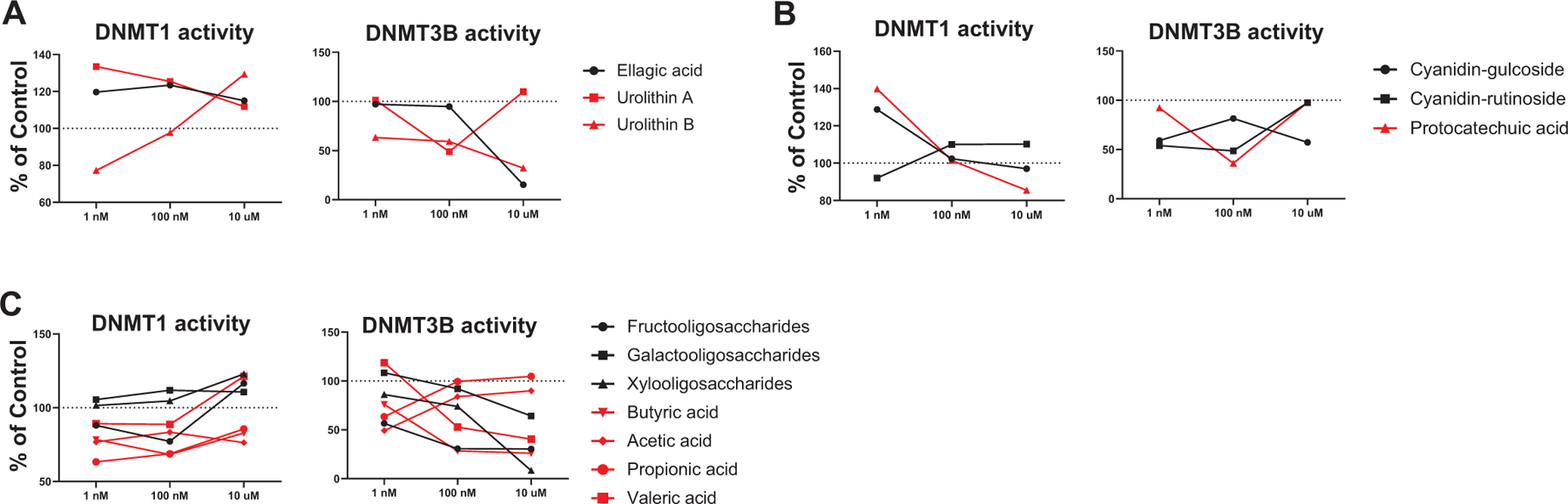
Effects of BRB phytochemicals and their gut bacterial metabolites on activities of DNMT1 and DNMT3B. (A) Ellagic acid, urolithin A, and urolithin B. (B) Cyanidin-glucoside, cyanidin-rutinoside, and protocatechuic acid. (C) Fructo-oligosaccharides, galacto-oligosaccharides, xylo-oligosaccharides, butyric acid, acetic acid, propionic acid, and valenic acid. Dimethyl sulfoxide was the control. Experiments were repeated three times and showed the same trends

When gut bacteria metabolize berry phytochemicals, the metabolites could be detected in circulating blood of mice and humans (Pan et al., 2015a, 2015b, 2017b, 2017c). We asked if BRB phytochemicals or their gut bacterial metabolites can suppress DNMT1 or DNMT3B activities. Ellagic acid and its gut bacterial metabolites, urolithin A and urolithin B, suppressed DNMT3B, though only urolithin B was inhibitory toward DNMT1 (Figure 5A). Similarly, cyanidin-glucoside and cyanidin-rutinoside as well as protocatechuic acid, their gut bacterial metabolite, was more effective at inhibiting the activity of DNMT3B than that of DNMT1 (Figure 5B). Interestingly, fructo-oligosaccharides, galacto-oligosaccharides, and xylo-oligosaccharides as well as their gut bacterial metabolites (the short-chain fatty acids butyric acid, acetic acid, propionic acid, and valeric acid) decreased the activity of both DNMT1 and DNMT3B, though the effect was more marked with DNMT3B (Figure 5C).

We reported that BRB-derived anthocyanins demethylate tumor suppressor genes by inhibiting DNMT1 and DNMT3B in colon cancer cells (Wang et al., 2013a). The current study suggests that other berry phytochemicals and their gut bacterial metabolites can also suppress DNMT1 and DNMT3B and that they must contribute collectively to the hypomethylation effects of whole BRBs. It should be noted that short-chain fatty acids function as HDAC inhibitors by activating free fatty acid receptor 2 and that loss of this receptor promotes colon cancer by epigenetic dysregulation of inflammation suppressors (Pan et al., 2017d, 2018). Results from the current study suggest an additional epigenetic mechanism that short-chain fatty acids use to suppress colorectal cancer.

## 4 | CONCLUSIONS

In addition to the importance of methylation in promoter regions for controlling gene expression, recent studies have suggested that methylation of intragenic and intergenic regions can change during physiological processes and disease (Kulis et al., 2013) and that intragenic methylation may play a major role in regulating cell-specific alternative promoters in gene bodies (Maunakea et al., 2010). BRBs hypomethylated promoter, intragenic, and intergenic regions annotated to genes in different signaling pathways, all of which are important for regulating immune function and inflammation, producing nitric oxide and reactive oxygen species, and promoting the progression of colorectal cancer. It is well known that phytochemical metabolites generated by gut bacteria regulate gut homeostasis (Bilotta & Cong, 2019). The current study showed that phytochemicals in BRBs, such as anthocyanins, ellagic acid, oligosaccharides, and their gut bacterial metabolites, suppress the activities of DNMT1 and DNMT3B. This finding suggests that other fruits and vegetables that contain those substances could well have the same ability to regulate DNA methylation. This hypothesis warrants further investigation.
